# Prognostic nutritional index as outcome predictor in patients with iliopsoas abscess

**DOI:** 10.1097/MD.0000000000031256

**Published:** 2022-10-28

**Authors:** Tomomasa Matsuo, Yasuhiko Fujita, Teruyoshi Amagai

**Affiliations:** a Department of Medicine, Tokunoshima Tokushukai General Hospital, Kagoshima, Japan; b Department of Radiology, Tokunoshima Tokushukai General Hospital, Kagoshima, Japan; c Department of Clinical Engineering, Faculty of Health Care Sciences, University of Jikei Health Care Sciences, Osaka, Japan.

**Keywords:** abscess volume, iliopsoas abscess (IPA), predictor, prognostic nutritional index (PNI)

## Abstract

Cases with iliopsoas abscess (IPA) in a single hospital-based cases were reviewed and compared with clinical profiles of published hospital-based IPA series. To verify usefulness of prognostic nutritional index (PNI) used to predict outcome and severity of IPA, this study was performed. This study consists of 2 parts: Study 1 – Case review of IPA series in a single hospital: 7 cases with IPA treated in a single hospital in sequential 5 years were collected (series 1) and their clinical profiles compared. Study 2 – Review of hospital-based literature: A search of the PubMed database from 1990 to the present was performed, using the Boolean expression ([Psoas OR iliopsoas] AND [abscess] AND [hospital-based]). Two hospital-based case series were collected. The clinical profiles of 2 series were compared with series 1 to draw predictive factors of outcome and deciding treatment modality, medical or surgical. Study 1 – Analyzing 7 IPA cases, average age was 76.7 years old (varying from 64 to 91) and the lifesaving rate was 86%. PNI < 45, calculated with serum albumin (Alb) and total lymphocyte count, and larger cumulative abscess volume (CAV) measured by computed tomography seem outcome predictors. Study 2 – Analyzing 2 hospital-based IPA series (series 2 and 3), series 2 reviewed isolated IPA cases without any comorbidities and series 3 reviewed IPA cases with cardiovascular disorders. Among 3 series including ours, series 1 showed oldest case and longer length of hospitalization. Series 3 showed the highest mortality among 3 because it collected IPA with cardiovascular comorbidities. PNI seems predictors of outcome and disease activity in patients with IPA and might indicate treated with surgical intervention.

## 1. Introduction

An iliopsoas abscess (IPA) is an infectious disease defined an abscess developed within iliopsoas muscle and rare. Its incidence has been reported 0.4 per 100,000.^[1,2]^ It must be considered life-threating when overwhelming sepsis occur and the average mortality rate varies from 6.7% to 18.9%.^[3,4]^ The IPA is classified into 2 subclasses by their causes, primary and secondary. The primary IPA is caused by disseminated organisms spreading from remote areas to iliopsoas region through blood or lymphatic streams. This is commonly associated with opportunistic conditions such as Diabetes Mellites,^[5]^ AIDS,^[5,6]^ or chronic renal failure.^[2]^ Secondary IPA is caused by infection spread to iliopsoas area from the adjacent organs directly such as entero-colonic perforation from Crohn’s fistulae,^[7]^ spinal tuberculosis,^[8]^ artificial medical devices of aortic endograft^[9]^ or hip arthroplasty,^[10]^ and infectious sacroiliitis.^[11]^

The causative organisms are reported that *Staphylococcus aureus* (*S aureus*) is the most common pathogen followed by *Escherichia coli*.^[5]^ The frequent 3 symptomatic presentations are denominated psoas triad, which includes fever, flank or back pain, and psoas sign observed as pain brought on by extension of the hip.^[12]^ IPA is mainly observed in middle age.^[5]^ We experienced relatively older patient with IPA among published case reports but was dead instead of intensive medical treatment. To save patients with IPA, using literature reviewing, we would find predictors of severity of IPA using previously reported and poor outcome by 2 methods: one was to compare clinical profiles of our experienced 7 cases to verify usefulness of prognostic nutritional index (PNI) calculated by serum albumin (Alb) and total lymphocyte count (TLC) used to predict outcome and severity of IPA, another was to compare our series with previously published hospital-based IPA series.

### 1.1. Aims

To verify usefulness of Prognostic nutritional index (PNI) used to predict outcome and severity of IPA.

## 2. Methods

### 2.1. Study 1: Case review of IPA series in a single hospital

We found 7 cases with IPA in the last 5 years between 2016 and 2020 searching electric records in our hospital. Their clinical profiles were compared to know the predictors of outcome in patients with IPA. The collected data included the follows: demographics of age, sex, symptoms and signs, laboratory data at admission including white blood cells, total lymphocytes (TLC), Alb, C-reactive protein, PNI^[13]^ calculated by the equation of [Alb (g/dL) × 10 + 0.005 × TLC (cells/mm^3^)], bacteriology of blood and urine if existed, computed tomography imaging to diagnose and calculate cumulative abscess volume of IPA (cm^3^) (cumulative IPA abscesses volume in each case: CAV) figuring all IPA volumes in each case, and the length of stay in hospital.

### 2.2. Study 2: Review of hospital-based literature

A search of the PubMed database from 1990 to the present was performed, using the Boolean expression ([Psoas OR iliopsoas] AND [abscess] AND [hospital-based]). We excluded single case reports. We included case series written in English language, and case series written in non-English language were include when the information of interest could be derived from the abstracts or from the manuscript itself. Then, as 2 hospital-based case series were collected, the clinical profiles of included these series were compared with our series to draw the possibility of predictability for the severity and selection of treatment modality, medical or surgical.

### 2.3. Ethical review

This study was approved by the Ethic Committee of the studied hospital. The ethic committee approval number is 20-03. From the naturality of the study, the opt-out method was applied and no refusals was stated.

### 2.4. Consent

The written informed consent to publication was not taken as the patient is sufficiently anonymized according to the ICMJE guidelines and when publication is approved by the authors’ ethics committee.

## 3. Results

### 3.1. Study 1

**Figure 1.**
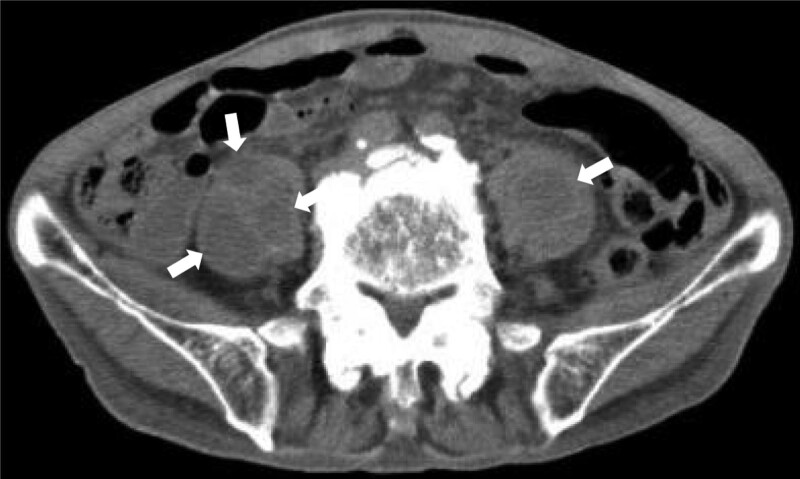
The abdominal computed tomography (CT) at lower level of 5th lumbar in case 1 of series 1. The arrows show multiple abscesses in bilateral psoas muscles.

**Figure 2.**
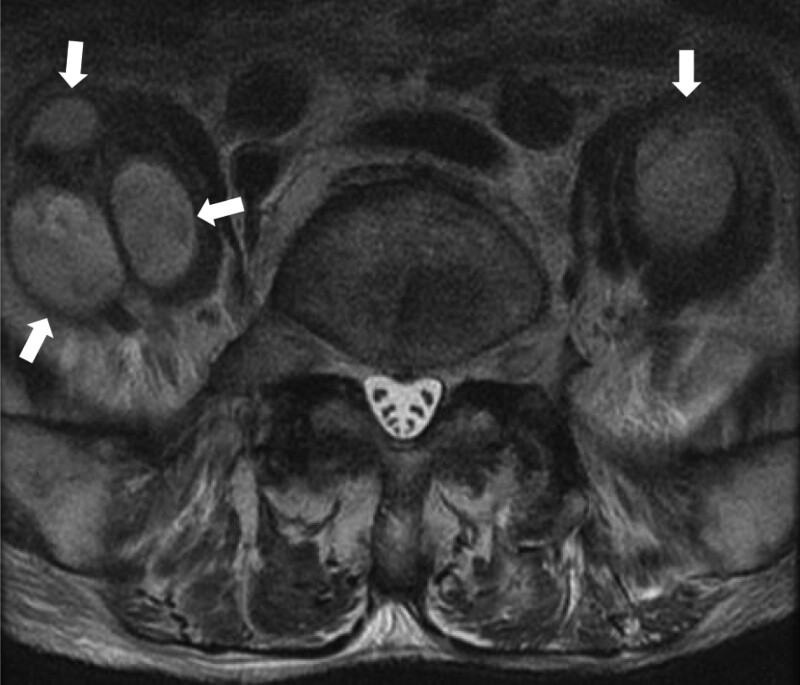
The magnetic resonance imaging (MRI), coronal T2-weighted image in case 1 of series 1. The arrows show in this MRI show high intensity-imaging of multiple abscesses in bilateral psoas muscles surrounded by low intensity capsules.

Among 7 cases with IPA experienced in our hospital during the last 5 years, the average age was 76.7 years old (varying from 64 to 91). The lifesaving rate was 86% (Table 1). The average values of laboratory data were all identical to moderate to severe infectious diseases (Table 2). The radiological studies of case 1 were shown (Fig. 1: abdominal computed tomography, Fig. 2: Magnetic Resonance Imaging). The bacteriology of culture materials of blood and urine showed infections of *S aureus* in 2 cases’ blood and *E coli* in 3 cases’ urine (Table 1). As such, these 2 cases with *S aureus* were septicemic and 1 of 2 was male, the oldest and dead instead of medical treatment (Table 1). Particularly in PNI, the oldest case had the smallest figure. In addition, the average CAV was 22.1 cm^3^ (ranging from 2 to 101 cm^3^). Among all cases, case 1 in Table 1 showed smallest PNI and the largest CAV and was not survived regardless of medical treatment. This result might mean that smaller PNI and the larger CAV might be predictor of poor outcome and surgical indicator as such treated by surgical interventions.

**Table 1:** Comparison of clinical characteristics of the present case and the other 6 IPA cases experienced in our hospital.

Cases	Age (yrs)	Sex	Co-morbidities	Symptoms	Bacteriology blood (urine)	LOS (d)	Outcome
1	91	Male	AD, scabies	Fever	MSSA	66	Dead
2	85	Male	thoracic aneurysm (stent replacement)	Fever, pain	MSSA	54	Survived
3	84	Male	AD, FPR	Fever, pain	ND	33	Survived
4	80	Female	-	Fever, pain	(*E coli*)	50	Survived
5	68	Male	HT, knee OA	Triad*	(*E coli*)	33	Survived
6	65	Male	UTI	Fever, pain	ND	102	Survived
7	64	Male	FPR	Fever	(*E coli*)	59	Survived

AD = Alzheimer’s disease, *E coli* = *Escherichia coli*, FPR = femoral prosthesis replacement, HT = hypertension, IPA = iliopsoas abscess, LOS = length of stay in hospital, MSSA = Methicillin-susceptible *Staphylococcus aureus*, ND = not detected, OA = osteoarthritis, UTI = urinary tract infection.

* triad: including fever, abdominal/ flank pain, psaos sign meaning pain brought on by extension of the hip.

**Table 2:** Comparison of laboratory data of 6 cases with IPA treated in a single hospital.

Cases	Age (yrs)	PNI	TLC (count/mm^3^)	Alb (mg/dL)	CRP (mg/dL)	CAV (cm^3^)
1	91	22.7	945	1.8	16.11	101
2	85	37.5	307	3.6	7.64	7
3	84	26.5	909	2.2	4.99	30
4	80	24.7	132	2.4	24.37	3
5	68	32.1	1023	2.7	19.04	8
6	65	33.1	418	3.1	20.09	4
7	64	40.8	562	3.8	9.12	2
Mean	76.7	31.1	613.7	2.8	14.5	22.1

Alb = serum albumin concentration, CAV = cumulative IPA abscesses volume, CRP = C-reactive protein, IPA = iliopsoas abscess, TLC = total lymphocyte count.

### 3.2. Review of hospital-based literature

Two hospital-based IPA series were reported in 2011 (series 2)^[2]^ and 2019 (series 3).^[3]^ Their patients’ profiles were shown (Table 3) to compare our series (series 1). Among these, patients with IPA involved in series 3 were associated with cardiovascular disorders including stent-graft/endograft infection of for abdominal aortic aneurysm, primary mycotic abdominal aortic aneurysm, and infective endocarditis. Another series (series 2) included isolated IPA cases without any surgical comorbidities. Among these 3 series, series 3 showed the highest mortality up to 40% probably because of severe co-morbidities with cardiovascular disorders compared with the other 2 series without them. The series 1 of ours showed older aged by 15 years and longer length of stay in hospital by 30 days comparing series 2.

**Table 3:** Comparison of clinical profiles in 3 hospital-based IPA series.

Hospital-based IPA series	Country	Type of reports	No. of cases	Average age	Male	Primary/secondary		Surgical treatment	Mortality	LOS, (d)
						Primary	Secondary			
Series 1, presented series	Japan	Hospital-based	7	76.6	86%	29%	71%	0%	14%	57
Series 2, ref. 2	Taiwan	Hospital-based	9	60.6	16%	56%	44%	11%	11%	26
Series 3, ref. 3	Taiwan	Hospital-based	15	63.2	80%	100%	0%	73%	40%	33

All data was expressed in %, except an average age expressed in years old.

LOS = length of stay in hospitals.

## 4. Discussion

IPA has been reported more common in middle aged,^[5]^ mostly associated with compromised conditions such as Diabetes Mellites, HIV infection, cancer, and steroid users. Hyperkeratotic scabies as comorbidity in the present case seems another cause of compromised infection. Hyperkeratotic scabies has been reported that scabies mites secrete complement inhibitors into burros, which potentially facilitates staphylococcal infections and subsequent invasive *Staphylococcus aureus* bacteremia (SAB) and sepsis.^[14]^ The authors added their analyses the high 1-year mortality in patients with scabies with SAB than patients without SAB (26% vs 8%, *P* = .002). This context seems consistent with our experienced case with scabies with SAB. From these observations, we could draw clinical attention when patients with bacterial infections such as IPA with SAB and scabies must be high mortality.

### 4.1. Prognostic nutritional index as a surrogate indicator of immunocompromised host

From stand point of view of laboratory profile, PNI has been reported as strong predictor of outcome in patients with various malignant and benign diseases as shown in series 1.^[15]^ PNI was originally developed by Buzby et al^[16]^ in 1980 to predict outcome in patients with gastrointestinal surgery. It is calculated by the equation of


$$\begin{array}{l} \left[=15816.6\times Alb0.78\times TSF0.22\times Tf5.8\times DH\right] \end{array}$$

Alb: serum albumin concentration (g/L), TSF: triceps skin folds (mm), Tf: transferrin (mg/dL), DH: delayed-type hypersensitivity skin test (spot forming units: SFU).

It was followed by the similar but far easier equation for the same purpose developed by Onodera T, et al in 1984.^[13]^ It is calculated by the equation mentioned in the method part. The later PNI (Onodera’s PNI) has been proved high prognostic value and well validated in various diseases.^[15,17,18]^ It has been showed to predict not only outcome but disease activity.^[19–21]^ In our study, PNI also seems to show outcome and disease activity because outcome must be associated with disease activity as two might be both sides of mirror of disease. The cutoff values of PNI varies according to various diseases. In this study, we used cutoff value at 45 as it was similarly in patients with infectious complication^[21]^ as IPA in this study. All cases of our series had PNI < 45. This might be interpreted that all 7 cases might be identified as severe infectious disease. However, the mortality rate is as low as 14% and the cutoff value for predicting outcome of patients with IPA must be examined. To our knowledge, it varies widely from 19.5 as the smallest in patients with acute kidney injury^[22]^ to 57 as the largest in extensive-stage small cell lung cancer.^[23]^ Under these circumstances with wide variation, the cutoff value for IPA cases must be determined using enough number of subjects in the further investigations.

### 4.2. Surgical interventions for IPA patients with larger abscesses volume

The treatment strategy consists of medical with antibiotics and surgical drainage. Among our experienced 7 cases (Table 1), all was treated medical without surgical drainage. From the results of study 1, the oldest case in series 1 had to be treated surgically because his PNI was the smallest and CAV was the largest (Table 2). As we have experienced the other 6 cases that were all treated medically and survived (Table 2), their IPA total volume were calculated retrospectively using CT images. All 6 IPA cases (case 2–7) showed IPA volume ≤ 30 cm^3^, whereas > 100 cm^3^ in case 1. This might mean that IPA volume is surrogate predictor of the severity and outcome. To extend this observational results in general, CAV > 100 cm^3^ in IPA cases also must be considered to treat with surgical intervention, unless patients have unstable hemodynamics or bleeding tendency (thrombocytopenia or prolonged prothrombin time/activated partial thromboplastin time).^[3]^ From aspect of CT measurement, CT-volumetric analysis seems surrogate predictor of severity of IPA. In addition, as the result of study 2, cardiovascular comorbidities in patients with IPA might also be poor outcome predictor. This might result from hypodynamic status in these comorbidities. However, as no data on hemodynamic status were written such as ejection fraction using echocardiography or serum brain natriuretic peptide concentration, this must be examined by the further studies.

### 4.3. Strength and limitations of this study

We would emphasize that this is the first to verify the usefulness of PNI in patients with IPA to our best knowledge. We added applicable range of PNI to the IPA by showing its usefulness in our series. We must warrant several limitations in this study. First, the number of IPA cases involved in our series was limited and it was difficult to draw the conclusive findings for CAV, because CAV is a novel method to evaluate the severity of abscess forming diseases such as IPA. Second, searching the hospital-based series of IPA series through PubMed engine, we found only 2 series except ours and both were reported from Taiwan. It must be limited to apply to complicated IPA cases with cardiovascular comorbidities with poor outcome only in Japanese and Taiwanese until this is found also in the other races. Third, in our series, surgical interventions were not included. The surgical strategy might be considered to severe IPA cases with low scores of PNI. However, it was not able to conclude because we did not. It must be examined in the further studied.

## 5. Conclusion

PNI seems predictors of outcome and disease activity in patients with IPA and might indicate treated with surgical intervention.

## Acknowledgments

We thank Enago (www.enago.tw) for the English language review of this manuscript.

## Author contributions

**Conceptualization:** Tomomasa Matsuo.

**Data curation:** Tomomasa Matsuo.

**Formal analysis:** Tomomasa Matsuo, Teruyoshi Amagai.

**Investigation:** Tomomasa Matsuo, Yasuhiko Fujita, Teruyoshi Amagai.

**Methodology:** Tomomasa Matsuo, Yasuhiko Fujita, Teruyoshi Amagai.

**Project administration:** Tomomasa Matsuo, Teruyoshi Amagai.

**Supervision:** Teruyoshi Amagai.

**Writing – original draft:** Tomomasa Matsuo.

**Writing – review & editing:** Yasuhiko Fujita, Teruyoshi Amagai.
